# Lack of galectin-3 modifies differentially Notch ligands in bone marrow and spleen stromal cells interfering with B cell differentiation

**DOI:** 10.1038/s41598-018-21409-7

**Published:** 2018-02-22

**Authors:** Felipe Leite de Oliveira, Sofia Nascimento dos Santos, Lauremilia Ricon, Thayse Pinheiro da Costa, Jonathas Xavier Pereira, Camila Brand, Marise Lopes Fermino, Roger Chammas, Emerson Soares Bernardes, Márcia Cury El-Cheikh

**Affiliations:** 1 Laboratório de Proliferação e Diferenciação Celular, Instituto de Ciências Biomédicas (ICB), Universidade Federal do Rio de Janeiro, Rio de Janeiro, RJ Brazil; 2 Centro de Radiofarmácia, Instituto de Pesquisas Energéticas e Nucleares (IPEN), São Paulo, SP Brazil; 3 Departamento de Clínica Médica, Faculdade de Ciências Médicas, Universidade Estadual de Campinas, Campinas, SP Brazil; 4 Programa de Pós-Graduação em Ciências Morfológicas, Instituto de Ciências Biomédicas, Universidade Federal do Rio de Janeiro, Rio de Janeiro, RJ Brazil; 5 Programa de Pós-Graduação em Anatomia Patológica, Faculdade de Medicina, Universidade Federal do Rio de Janeiro, Rio de Janeiro, RJ Brazil; 6 Faculdade de Ciências Farmacêuticas de Ribeirão Preto, Universidade de São Paulo, Ribeirão Preto, SP Brazil; 7 Laboratório de Oncologia Experimental e Instituto do Câncer do Estado de São Paulo, Faculdade de Medicina, São Paulo, Brazil

## Abstract

Galectin-3 (Gal-3) is a β-galactoside binding protein that controls cell-cell and cell-extracellular matrix interactions. In lymphoid organs, gal-3 inhibits B cell differentiation by mechanisms poorly understood. The B cell development is dependent on tissue organization and stromal cell signaling, including IL-7 and Notch pathways. Here, we investigate possible mechanisms that gal-3 interferes during B lymphocyte differentiation in the bone marrow (BM) and spleen. The BM of gal-3-deficient mice (Lgals3^−/−^ mice) was evidenced by elevated numbers of B220^+^CD19^+^c-Kit^+^IL-7R^+^ progenitor B cells. In parallel, CD45^−^ bone marrow stromal cells expressed high levels of mRNA IL-7, Notch ligands (Jagged-1 and Delta-like 4), and transcription factors (Hes-1, Hey-1, Hey-2 and Hey-L). The spleen of Lgals3^−/−^ mice was hallmarked by marginal zone disorganization, high number of IgM^+^IgD^+^ B cells and CD138^+^ plasma cells, overexpression of Notch ligands (Jagged-1, Delta-like 1 and Delta-like 4) by stromal cells and Hey-1. Morever, IgM^+^IgD^+^ B cells and B220^+^CD138^+^ CXCR4^+^ plasmablasts were significantly increased in the BM and blood of Lgals3^−/−^ mice. For the first time, we demonstrated that gal-3 inhibits Notch signaling activation in lymphoid organs regulating earlier and terminal events of B cell differentiation.

## Introduction

Galectin-3 (gal-3) is a β-galactoside-binding protein which controls cell–cell and cell–extracellular matrix interactions modulating cellular proliferation, differentiation, homing and survival^1^. Several types are responsible for its production, including monocytes, macrophages, granulocytes, and activated T and B lymphocytes^2^. In the course of conventional B cell differentiation, gal-3 shows a potent inhibitory role by regulating cell fate decisions to memory phenotype or plasma cell generation^3,4^. In non-conventional peritoneal B1 lymphocytes biology, gal-3 also plays regulatory roles in the differentiation of both B1a and B1b cells into plasma cells by IL-5 and Blimp-1 signaling-dependent manner^5^. Clearly, gal-3 interferes with B cell compartments in distinct lymphoid organs^4–8^. However, the mechanisms that correlate gal-3 with molecular pathways during bone marrow B lymphopoiesis, peripheral mobilization and settlement mainly in the spleen, are poorly understood.

In the bone marrow of adults, B lymphocytes are generated continuously under stromal and cytokine control, including IL-7^9,10^. A common lymphoid precursor differentiate into B220^+^CD19^−^c-Kit^+^IL-7R^+^IgM^−^IgD^−^ pre-pro B cells and subsequently, these cells originate B220^+^CD19^+^c-Kit^+^IL-7R^+^IgM^−^IgD^−^ pro B cell and B220^+^CD19^+^c-Kit^−^IL-7R^+^IgM^−^IgD^−^ pre B cells. The B220^+^CD19^+^c-Kit^−^IL-7R^−^IgM^+^IgD^−^ immature B cells receive signals to home to secondary lymphoid organs, such as the spleen, becoming IgM^+^IgD^+^ follicular (FO) or marginal zone (MZ) B cells^11^.

There are several biological mechanisms that determine the B cell fate decision in the bone marrow, peripheral distribution and settlement in the spleen. In this context, Notch signaling pathways appear as extreme biological relevance^10,12^. Distinct members of Notch family signaling are involved with the homing of immature B cells^13^. The Notch ligands, including Delta-like (Dll) and Jagged (Jag), are largely expressed by splenic endothelial cells favoring the differentiation of MZ B lymphocytes over the FO B lymphocytes^14,15^.

The promptly responses against blood antigens in the spleen is directly associated to histological architecture integrity that drives the terminal differentiation of B cells. Cell fate choices to follicular or marginal zone B cell phenotype are dependent on signaling by the B cell receptor, Notch pathways, and other receptors that include B cell-activating factor and nuclear factor-kappa B mechanisms^11,16^. Recently, we showed significant disturbances on B cell niches in the spleen and mesenteric lymph nodes of gal-3 deficient mice (Lgals-3^−/−^ mice) associated with atypical plasma cell generation during chronic schistosomiasis^6,7^. Clearly, the organization of functional niches is responsible for stability of the spleen by regulating local amplification and retention of B cells. However, the immunomodulatory role of gal-3 interfering with molecular pathways driving B cell differentiation is poorly understood, in both lymphoid organs: bone marrow and spleen.

Here, we investigated whether gal-3 interferes with a Notch signaling pathways that control the bone marrow B lymphopoiesis and terminal B cell differentiation in the spleen. For the first time, it was demonstrated that stromal cells in the bone marrow and spleen of Lgals3^−/−^ mice expressed higher levels of Notch ligands than wild type (Lgals3^+/+^) mice. These events were directly correlated with increased levels of IL-7 in the bone marrow justifying the intense B cell proliferation, as well as, high number of circulating IgM^+^IgD^+^ B cells and B220^+^CD138^+^ CXCR4^+^ plasmablasts indicating spleen disorganization.

## Results

### Bone marrow B lymphopoiesis is inhibited in Lgals3^−/−^ mice

**Figure 1:**
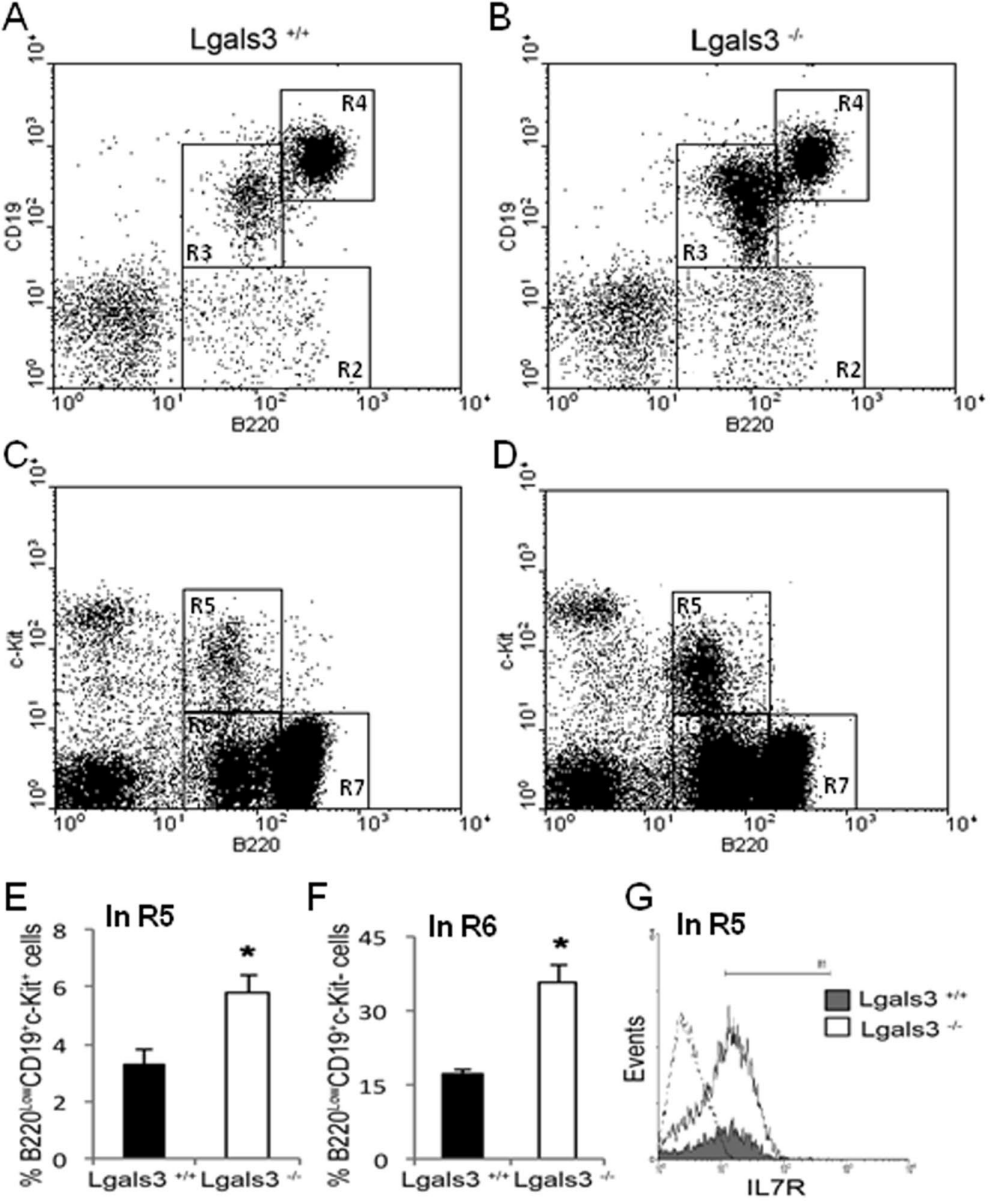
Phenotype of B cell subpopulations in the bone marrow. B cell subtypes were subdivided in gates R2: B220^low^CD19^−^ cells, R3: B220^low^CD19^+^ cells, R4:- B220^high^CD19^high^, R5: B220^low^c-Kit^low^ cells, R6: B220^low^c-Kit^−^ cells, R7: B220^high^c-Kit^+^ were quantified in Lgals3^+/+^ (**A** and **C**) and Lgals3^−/−^ mice (**B** and **D**).Triple stained B220^low^CD19^+^c-Kit^+^ progenitor B cells (**E**) and B220^low^CD19^+^c-Kit^−^ B cells (**F**) were also measured in the bone marrow of Lgals3^+/+^ (black bars) and Lgals3^−/−^ (white bars) mice. (**G**) Histograms show quadruple staining and quantity of B220^+^CD19^+^c-Kit^+^IL-7R^+^ B cells in the bone marrow of Lgals3^+/+^ (gray histogram) and Lgals^−/−^ (white histogram) mice; Dashed histogram: Ig-isotype control. *n* = 5 mice per group. (*) Indicates p < 0.05. Dot plot graphs are representative of each mice group.

Gal-3 inhibits terminal differentiation of B lymphocytes into plasma cells^3,4^. However, its mechanistic role in B lymphopoiesis has not been investigated so far. To elucidate this question, we first compared the kinetics of B lymphocyte production in the bone marrow of Lgals3^+/+^ and Lgals3^−/−^ mice. First, the lymphocyte-gate was defined for both mice and the percentage of gated cells was increased in Lgals3^−/−^ mice (data not shown). In these mice, the bone marrow B220^+^CD19^−^ cells and B220^low^CD19^low^ cells were both increased when compared with Lgals3^+/+^ mice (Fig. 1A and B, gate R2 and R3 respectively). On the other hand, immature B220^high^CD19^high^ B cells were perceptibly reduced in the bone marrow of Lgals3^−/−^ mice (Fig. 1A and B, gate R4). In fact, B220^low^c-Kit^+^ cells and B220^low^c-Kit^−^ cells were increased in the bone marrow of Lgals3^−/−^ mice (Fig. 1C and D, gate R5 and R6 respectively). Corroborating, bone marrow B220^high^c-Kit^−^ cells were reduced in the absence of gal-3 (Fig. 1C and D, gate R7). Percentage and absolute number of B cell subpopulations analyzed in each gate (R2-R7) are described in Tables 1 and 2, respectively.

**Table 1:** Percentage of B cell subpopulations in the bone marrow of Lgals3^+/+^ and Lgals3^−/−^ mice.

*B cell subpopulation*	*Lgals3*^+/+^ (% of cells)	*Lgals3*^−/−^ (% of cells)
B220^+^CD19^−^ (gate R2)	1.35 ± 0.2	2.31 ± 0.41
B220^low^CD19^low^ (gate R3)	2.32 ± 0.36	10.66 ± 1.98
B220^high^CD19^high^ (gate R4)	9.32 ± 1.12	8.85 ± 1.27
B220^low^c-kit^+^ (gate R5)	0.49 ± 0.19	1.29 ± 0.22
B220^low^c-kit^−^ (gate R6)	3.20 ± 0.33	5.11 ± 0.85
B220^high^c-kit^−^ (gate R7)	7.21% ± 1.1	8.85% ± 0.99

Data are plotted as means obtained from three independent experiments.

**Table 2:** Absolute number of B cell subpopulations in the bone marrow of Lgals3^+/+^ and Lgals3^−/−^ mice.

*B cell subpopulation*	*Lgals3*^+/+^ (x 10^5^ cells)	*Lgals3*^−/−^ (x 10^5^ cells)
B220^+^CD19^−^ (gate R2)	2.92 (±0.31)	2.63 (±0.41)*
B220^low^CD19^low^ (gate R3)	3.64 (±0.56)	11.98 (±1.85)***
B220^high^CD19^high^ (gate R4)	1.44 (±0.28)	1.01 (±0.12) **
B220^low^c-kit^+^ (gate R5)	4.96 (±0.71)	5.81 (±0.92)
B220^low^c-kit^−^ (gate R6)	4.12 (±0.58)	10.89 (±1.67)***
B220^high^c-kit^−^ (gate R7)	11.17 (±1.59)	6.58 (±1.02)**

Data are plotted as means obtained from three independent experiments. (*) indicates p ≤ 0.05, (**) indicates p ≤ 0.01 and (***) indicates p < 0.001.

The cell cycle in the total bone marrow cells was substantially modified in the absence of gal-3, with a high percentage of cells in the S and G2/M phase of the cell cycle, compatible with a more proliferative status (Supplementary Fig. 1). Triple phenotypic analysis revealed that B220^low^CD19^low^c-Kit^+^ and B220^low^CD19^low^c-Kit^−^ progenitor B cell subpopulations were significantly increased in Lgals3^−/−^ in comparison with Lgals3^+/+^ mice (Fig. 1E and F, respectively). Quadruple phenotypic evaluation indicated that B220^low^CD19^low^c-Kit^+^IL7R^+^ were substantially modified in the absence of gal-3 (Fig. 1G). Taken together, these date indicate that gal-3 plays a role in the expansion of IL-7R+ progenitors phenotypically characterized as B220^low^CD19^low^c-Kit^+^ IL7R^+^ in the bone marrow.

### High levels of B cell precursors were associated with IL-7 and JAG1 expression in bone marrow of Lgals3^−/−^ mice

**Figure 2:**
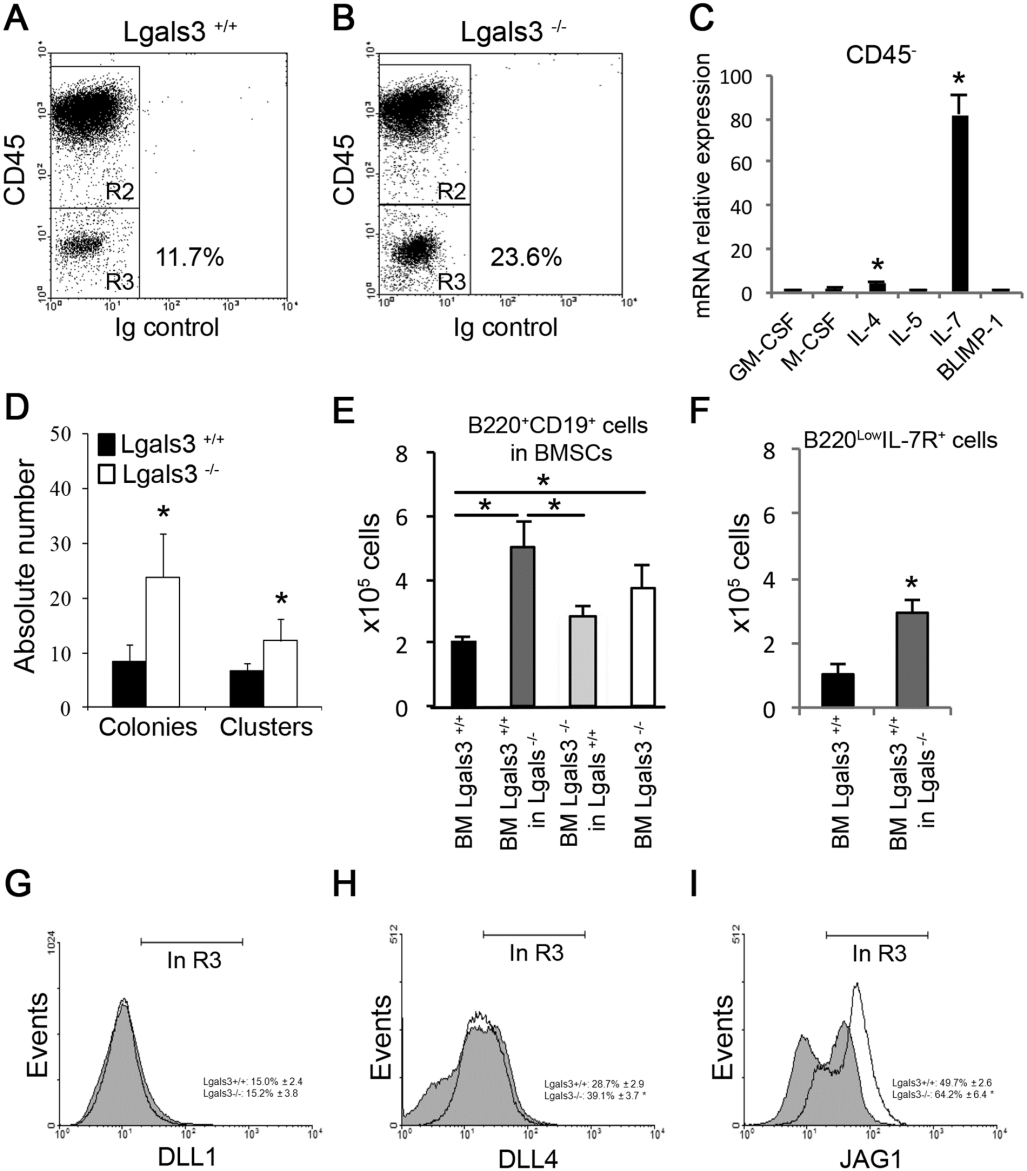
Characterization of bone marrow stromal cells (BMSCs). CD45^−^ BMSCs (gate R3) of Lgals3^+/+^ (**A**) and Lgals3^−/−^ mice (**B**). (**C**) The relative expression of genes involved with hematopoietic events was compared between CD45^−^ BMSCs of Lgals3^+/+^ and Lgals3^−/−^ mice (Lgals3^−/−^ per Lgals3^+/+^). (**D**) Total bone marrow cells were cultured in the methylcellulose system containing B cell-enriched growth factors: colonies (maximum 50 cells) and clusters (minimum 50 cells). (**E**) Coculture systems using different strategies to quantify B220^+^CD19^+^ B cells: BM Lgals3^+/+^ (culture of total bone marrow cells of Lgals3^+/+^); BM Lgals3^+/+^ in Lgals3^−/−^ (bone marrow cells of Lgals3^+/+^ mice cultured in BMSCs of Lgals3^−/−^); BM Lgals3^−/−^ in Lgals3^+/+^ (bone marrow cells of Lgals3^−/−^ cultured in Lgals3^+/+^ BMSCs); BM Lgals3^−/−^ (culture of total bone marrow cells of Lgals3^+/+^). (**F**) Number of B220^low^IL-7R^+^ cells after 1 week using similar co-culture strategy. (**G**–**I**) Histograms represent the expression of Delta-like 1 (DLL1), Delta-like 4 (DLL4) and Jagged-1 (JAG1) by CD45^−^ BMSCs (gated in R3, A and B). *n* = 5 mice per group. (*) Indicates p < 0.05. Dot plot graphs are representative of each mice group.

Since IL-7-expressing B lymphocyte precursors are partly dependent on stromal cells to continue the cellular differentiation process^17^, we next evaluated the protein levels of CD45 in stromal cells from Lgals3^+/+^ and Lgals3^−/−^ mice (Fig. 2A e 2B). We found an increase percentage of CD45^−^ bone marrow stromal cells in Lgals3^−/−^ mice compared with the Lgals3^+/+^ (Fig. 2A,B, in R3). The pattern of cytokines involved with B cell differentiation was analyzed on these CD45^−^ stromal cells from both experimental groups of mice. The relative expression of mRNA IL-7 and of IL-4 increased 40-fold and 5-fold, respectively, in the bone marrow stromal cells of Lgals3^−/−^ mice in comparison to Lgals3^+/+^ mice (Fig. 2C). However, the relative expression of G-CSF, GM-CSF, IL-5 and Blimp-1 was similar between stromal cells of both groups of mice (Fig. 2C). To test the functionality of IL-7R in B cell precursors, we used the clonogenic assay adding recombinant IL-7 to quantify colonies and clusters derived from total bone marrow of both experimental groups. Colonies and clusters were significantly increased in Lgals3^−/−^ mice compared with Lgals3^+/+^ mice (Fig. 2D).

Next, we observed that B lymphocytes maturation was increased in Lgals3^−/−^ in comparison with Lgals3^+/+^ mice (Fig. 2E). In order to evaluate early interactions between B cells precursors and stromal bone marrow cells (BMSCs), we co-cultured B220^+^CD19^+^ lymphocytes cells of Lgals3^+/+^ on BMSCs of Lgals3^−/−^ mice and B220^+^CD19^+^ lymphocytes of Lgals3^−/−^ on BMSCs of Lgals3^+/+^ mice. We found that the number of B lymphocytes was increased when B cells precursors derived from Lgals3^+/+^ mice were co-cultured with Lgals3^−/−^ BMSCs (Fig. 2E) in comparison with Lgals3^+/+^ BMSCS. In contrast, no difference in the number of B lymphocytes maturation was observed when B cell precursors were co-cultured with Lgals3^+/+^ derived BMSCs regardless of B cell precursors background (Fig. 2E). In accordance, B220^low^IL-7R^+^ B from Lgals3^+/+^ mice cells were significantly increased when co-cultured with Lgals3^−/−^ stromal cells (Fig. 2F). Potential mechanisms were evaluated in the BMSCs of Lgals3^−/−^ mice, such as the expression of Notch receptors DLL1, DLL4 and JAG1, classical signaling pathway involved in bone marrow B cell development^12,18^. DLL1 and DLL4 protein levels were not modified in CD45^−^ cells derived from Lgals3^+/+^ and Lgals3^−/−^ mice (Fig. 2G and H). However, CD45^−^ cells from Lgals3^−/−^ mice expressed higher levels of JAG1 (Fig. 2I) in comparison with Lgals3^+/+^ mice. Values of percentage of cells expressing DLL1, DLL4, and JAG1 were described adjacent to respective histograms (Fig. 2G–I). The number of DLL4^+^ and JAG1^+^ cells in the bone marrow CD45- stromal cells was significantly increased in the absence of gal-3, suggesting that this protein controls B cell differentiation in a stromal dependent manner and, at least in part, interfering with IL-7 gene expression.

### Notch signaling pathway is increased in the bone marrow of Lgals3^−/−^ mice

**Figure 3:**
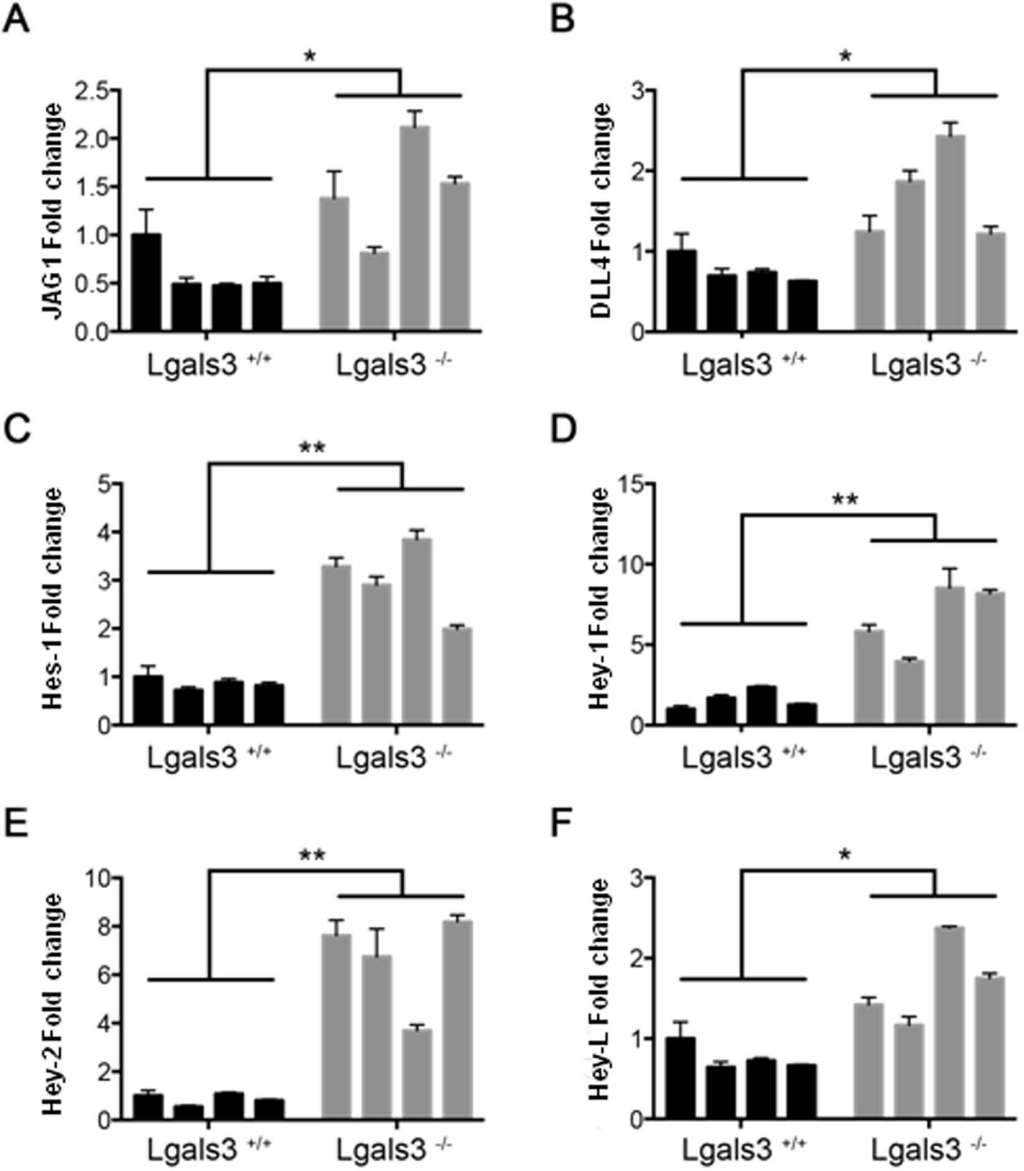
Notch signaling pathway in the bone marrow of Lgals3^−/−^ mice. Expression of the Notch signaling pathway components in total bone marrow cells of Lgals3^+/+^ (black bars) and Lgals3^−/−^ mice (gray bars). The expression levels of JAG1 (**A**) DLL4 (**B**) ligands was measured in Lgals3^+/+^ and Lgals3^−/−^ mice. Notch target transcription factors Hes-1 (**C**), Hey-1 (**D**), Hey-2 (**E**) and Hey-L (**F**) werealso evaluated in both mice groups. *n* = 4 mice per group. (*) Indicates p < 0.05.

Since gal-3 has been shown to regulate Notch signaling activation in angiogenesis, osteoblast differentiation and dendritic cells maturation^19–21^, we next evaluated the expression of the Notch signaling pathway component in total bone marrow cells obtained *ex vivo* from Lgals3^+/+^ and Lgals3^−/−^ mice. We observed that the expression levels of JAG1 and DLL4 ligands were increased in Lgals3^−/−^ mice compared with the Lgals3^+/+^ mice (Fig. 3A and B, respectively). Moreover, the higher expression of JAG1 and DLL4 in Lgals3^−/−^ mice was followed by an increased expression of the Notch target transcription factors Hes-1, Hey-1, Hey-2 and Hey-L (Fig. 3C–F respectively). These results strongly suggest that gal-3 inhibits Notch signaling activation in the bone marrow microenvironment.

### Splenic marginal zone is disturbed in the absence of gal-3

**Figure 4:**
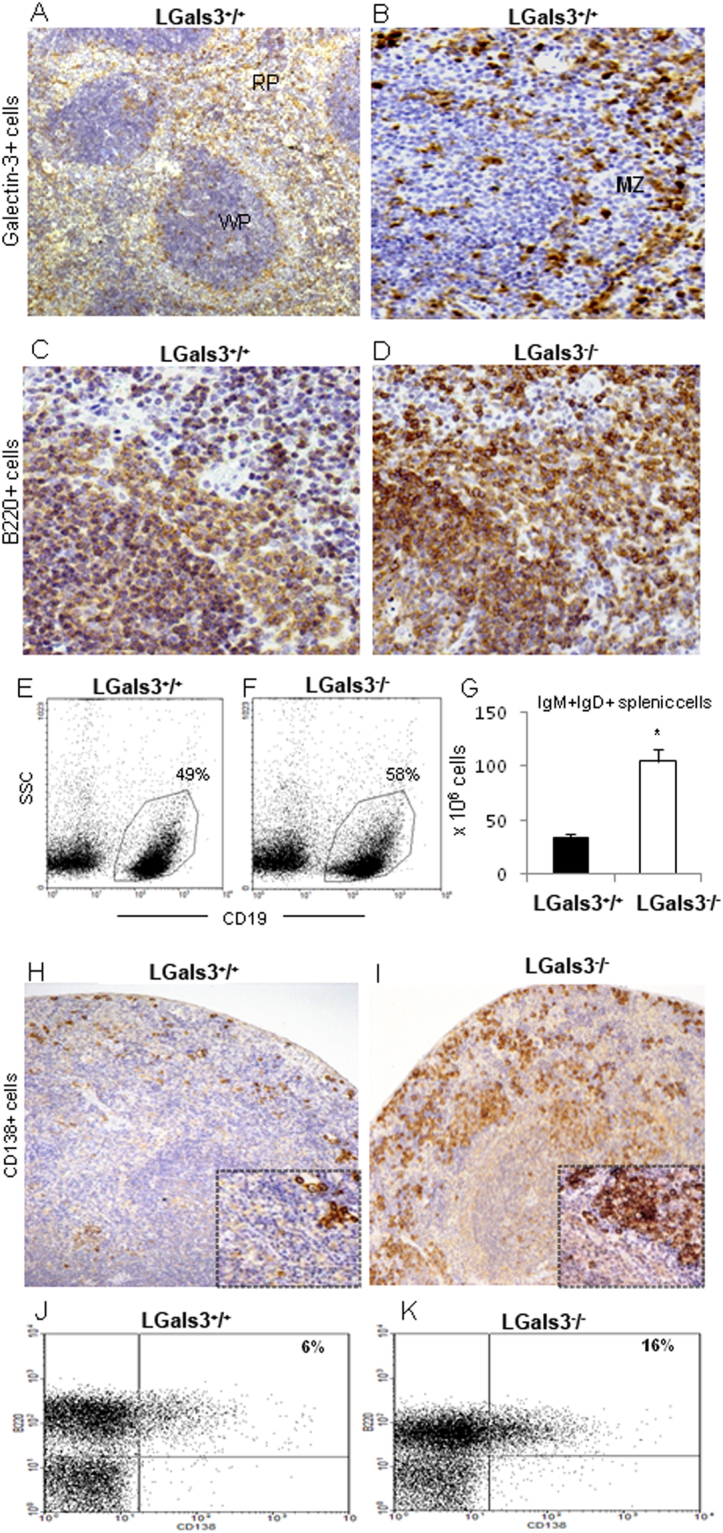
Histological analysis of the spleen. (**A**) Localization of galectin-3^+^ cells in the white (WP) and red pulp (RP). (**B**) In more details, galectin-3^+^ cells were located in the marginal zone (MZ). Both photomicrographs are representative of WT mice. B cell niches were hallmarked by B220^+^ B cells in MZ of Lgals3^+/+^ mice (**C**), but poorly identified in Lgals3^−/−^ mice (**D**). Identification of splenic CD19^+^SSC^low^ B cells in Lgals3^+/+^ (**E**) and Lgals3^−/−^ mice (**F**). (**G**) Quantity of IgM^+^IgD^+^ B cells identified by flow cytometry. CD138^+^ plasma cells were located in the spleen of Lgals3^+^^/^^+^ (**H**) and Lgals3^−/−^ mice (**I**). Flow cytometry analysis revealed B220^+^CD138^+^ plasmablasts in Lgals3^+/+^ and Lgals3^−/−^ mice (**J** and **K**, respectively). *n* = 5 mice per group. Magnification: (**A**,**B**) 100×; (**C**,**D**) 200×; (**H**,**I**) 100×. (*) Indicates p < 0.05.

So far, our results demonstrated that in the absence of gal-3, the number of B cells precursors is increased in the bone marrow. Since the spleen is the major receptor organ of immature B cells we aimed to study how the increased number of B cell precursors is interfering with the function of the spleen. Firstly we investigated the distribution of gal-3 in the spleen of Lgals3^+/+^ mice and detected positive cells within the lymphoid follicles and forming cord-like structures in the edge of white and red pulp (Fig. 4A and B, respectively). A quantitative analysis showed that approximately 15% of follicular cells expressed gal-3, while approximately 25% of extra-follicular cells were positive to gal-3 (Supplementary Fig. 2A). Furthermore, a conventional histological analysis revealed that the marginal zone from Lgals3^−/−^ mice was poorly defined in comparison with Lgals3^+/+^ mice (Supplementary Fig. 2B and C). Moreover, staining for B220^+^ cells revealed that cells were not constricted to the lymphoid follicle structure in Lgals3^−/−^ mice and were also found in the red pulp in contrast to the conventional distribution observed in Lgals3^+/+^ mice (Fig. 4C and D). These results suggest that the absence of gal-3 disrupt B cell niches in the spleen, affecting the normal pathway of B cell terminal differentiation. In fact, the number of IgM^+^IgD^+^ mature B cells was significantly increased in the spleen of Lgals3^−/−^ mice (Fig. 4E–G).

Splenic plasma cell niches were substantially disturbed in the absence of gal-3. In Lgals3^+/+^ mice, CD138^+^ plasma cells were typically found in the marginal zone of the spleen (Fig. 4H). However, in the absence of gal-3 CD138^+^ plasma cells were detected randomly distributed and clearly clustered throughout the spleen (Fig. 4I). Accordingly, the percentage of B220^+^CD138^+^ plasma blast-like cells was significantly increased in Lgals3^−/−^ mice (16%) in comparison with Lgals3^+/+^ (6%) (Fig. 4J and K).

### Lack of gal-3 increases B cell terminal differentiation through Notch signaling

**Figure 5:**
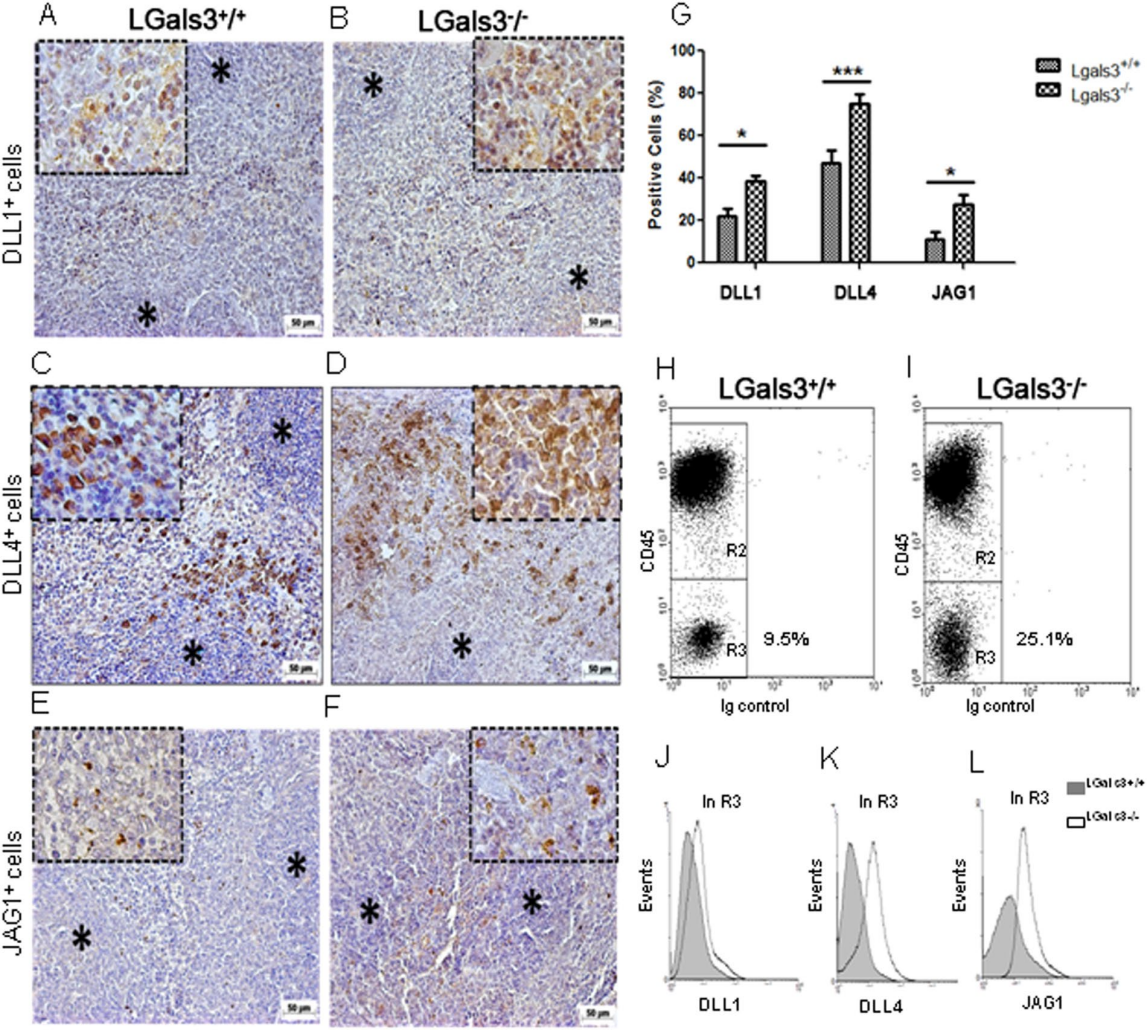
Localization of Notch-ligands expressing cells in the spleen. (**A**) DLL1^+^ cells in extrafollicular sites of Lgals3^+/+^ mice. (**B**) DLL1^+^ cells atypically distributed on Lgals3^−/−^ mice. (**C**) DLL4^+^ cells in well defined sites in Lgals3^+/+^ mice and (**D**) disorganized in Lgals3^−/−^ mice. (**E**) JAG1^+^ cells on extrafollicular regions of Lgals3^+/+^ mice and randomly distributed in Lgals3^−/−^ mice (**F**). (**G**) Percentage of splenic cells according positivity to DLL1, DLL4 and JAG1. Dot plot graphs by flow cytometry identify hematopoietic (CD45^+^) cells and stromal (CD45^−^) cells in both Lgals3^+/+^ and Lgals3^−/−^ mice (H and I, in R3, respectively). Histograms indicate the intensity of expression of DLL1 (**J**), DLL4 (**K**) and JAG1 (**L**) in both groups of mice. *n* = 5 mice per group. Magnification: (**A**–**F**) 100×; inserts 400×; DLL1 = Delta-like 1, DLL4 = Delta-like 4 and JAG1 = Jagged-1. (*) Indicates p < 0.05. (***) Indicates p < 0.01.

In the presence of gal-3, DLL1^+^ cells were preferentially observed on well-defined extrafollicular sites (Fig. 5A). In contrast, these cells were atypically found in within lymphoid follicles in the spleen of Lgals3^−/−^ mice (Fig. 5B). Similar properties were observed to DLL4^+^ splenic cells in Lgals3^+/+^ and Lgals3^−/−^ mice, with clear extrafollicular positivity in the presence of gal-3 and substantial compartmental disorganization in the absence of gal-3 (Fig. 5C and D, respectively). JAG1^+^ cells were also located on extrafollicular regions in the spleen of Lgals3^+/+^ mice whereas they were randomly distributed in Lgals3^−/−^ mice (Fig. 5E and F, respectively).

Clearly, DLL1^+^, DLL4^+^ and JAG1^+^ cells in Lgals3^−/−^ mice presented a diffuse pattern of distribution, in contrast to more focal organization on extrafollicular areas in the Lgals3^+/+^ mice. The percentage of these cells was measured in the spleen of both Lgals3^+/+^ and Lgals3^−/−^ mice. In the absence of gal-3, it was observed that DLL1^+^, DLL4^+^ and JAG1^+^ cells were numerically increased in comparison with respective controls (Fig. 5G).

To elucidate the cell subpopulation hallmarked by the expression of DLL1, DLL4 and JAG1, splenic cells were subdivided into according to positivity or negativity for CD45. CD45^−^ cells were significantly increased in the spleen of Lgals3^−/−^ mice when compared to Lgals3^+/+^ mice (Fig. 5H and I, in R3). These stromal cells showed increased levels of DLL1, DLL4 and JAG1 in Lgals3^−/−^ when compared with Lgals3^+/+^ mice (Fig. 5J–L). Altogether our results suggest a direct correlation between the exacerbated plasmacytogenesis in the absence of gal-3 with increased presence of Notch ligands in the splenic stromal cells.

**Figure 6:**
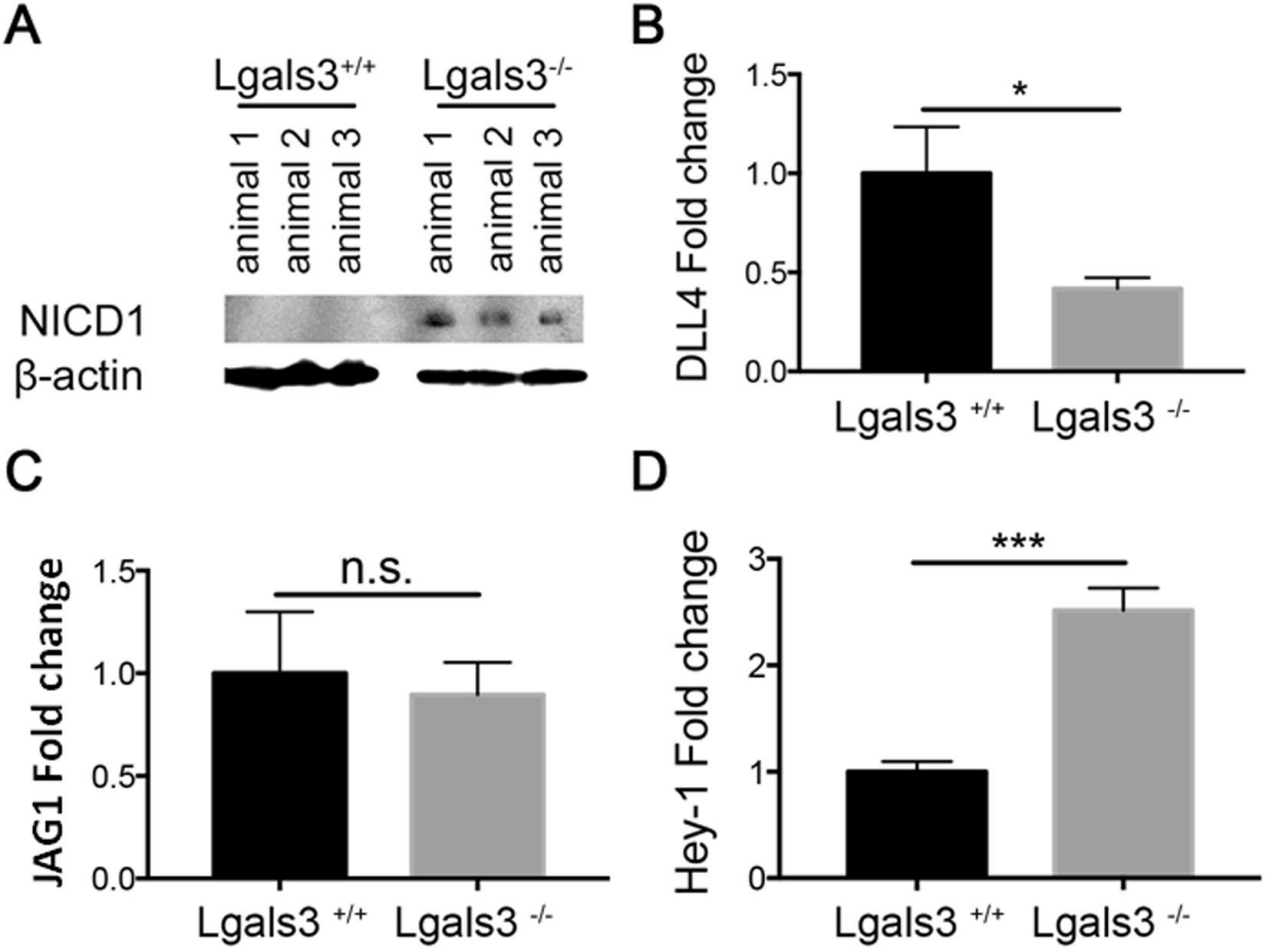
Notch target genes on B220^+^ cells sorted by FACS. (**A**) Notch-1 intracellular domain (NICD1) was significantly cleavage in Lgals3^−/−^ B220^+^ cells and DLL4 was more evident in Lgals3^+/+^ B220^+^ cells. β-actin was used as endogenous control. (**B**) mRNA levels of DLL4 in B220^+^ cells of Lgals3^+/+^ and Lgals3^−/−^ mice. (**C**) mRNA levels of JAG1 in B220^+^ cells of both mice groups. (**D**) mRNA levels of Notch target gene Hey-1 in B220^+^ cells of Lgals3^+/+^ and Lgals3^−/−^ mice. *n* = 5 mice per group. (*) Indicates p < 0.05. (***) Indicates p < 0.01.

To investigate molecular mechanisms of Notch signaling pathways disturbed in the splenic B lymphocytes of Lgals3^−/−^ mice, B220^+^ cells were sorted by FACS technology and Notch target genes were evaluated. In these Lgals3^−/−^ mice, a significant cleavage of the Notch-1 intracellular domain (NICD1) was observed on B220^+^ cells in comparison with B220^+^ cells of Lgals3^+/+^ mice (Fig. 6A). In contrast, RNA levels of DLL4 were significantly increased in B220^+^ cells of Lgals3^+/+^ mice when compared to Lgals3^−/−^ mice (Fig. 6B). No difference was observed in the RNA levels of JAG1 in both groups of mice (Fig. 6C). The release of NICD1 in B220^+^ cells from Lgals3^−/−^ was followed by up-regulation of the Notch target gene Hey-1 (Fig. 6D). These results suggest that the enhanced B-cell differentiation into plasma cells in the absence of gal-3 is regulated by the Notch signaling pathway.

### Gal-3 controls migration of IgM^+^IgD^+^ and CXCR4^+^CD138^+^B220^+^ cells

**Figure 7:**
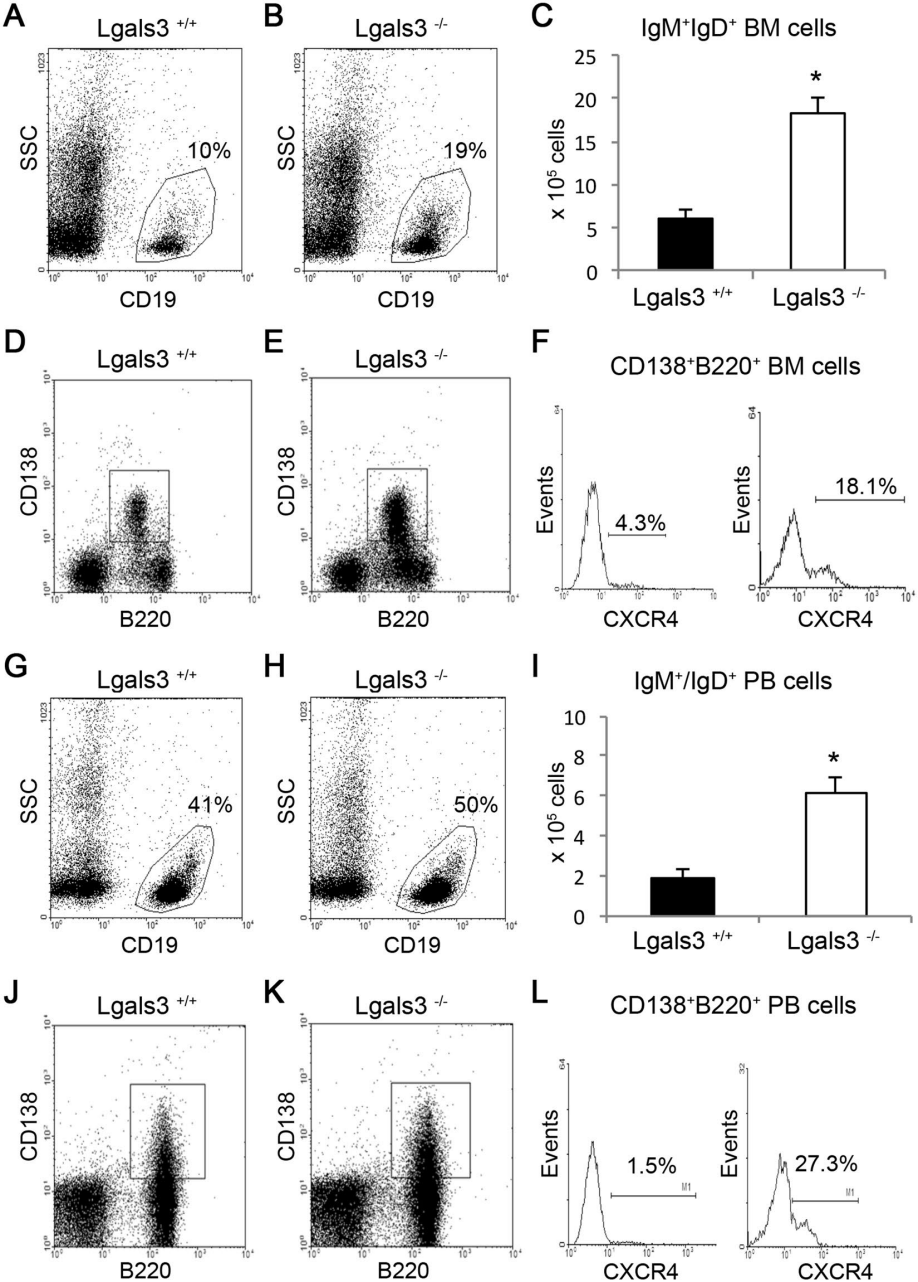
Kinetics of IgM^+^IgD^+^ and B220^+^CD138^+^CXCR4^+^ cell subpopulations in the absence of galectin-3. Percentage of total CD19^+^ B cells in the bone marrow of Lgals3^+/+^ (**A**) and Lgals3^−/−^ mice (**B**). (**C**) Absolute number of the bone marrow CD19^+^ B cells. Percentage of total B220^+^CD138^+^ B cells in the bone marrow of Lgals3^+/+^ (**D**) and Lgals3^−/−^ mice (**E**). (**F**) Percentage of B220^+^CD138^+^CXCR4^+^ plasmablasts in the bone marrow of both Lgals3^+/+^ and Lgals3^−/−^ mice. Percentage of total CD19^+^ B cells in the blood of Lgals3^+/+^ (**G**) and Lgals3^−/−^ mice (**H**). (**I**) Absolute number of CD19^+^ B cells in the bloodstream of both mice. Percentage of total B220^+^CD138^+^ B cells in the blood of Lgals3^+/+^ (**J**) and Lgals3^−/−^ mice (**K**). (**L**) Percentage of B220^+^CD138^+^CXCR4^+^ plasmablasts in the bloodstream of Lgals3^+/+^ and Lgals3^−/−^ mice. *n* = 5 mice per group. (*) Indicates p < 0.05.

To monitor the kinetics of B lymphocyte and plasma cell circulation, we quantified the number of IgM^+^IgD^+^ cells in the bone marrow and peripheral blood. In the bone marrow, the percentage of total CD19^+^ B cells and absolute number of IgM^+^IgD^+^ B cells were significantly increased in the absence of gal-3 (Fig. 7A–C). The percentage of B220^+^CD138^+^CXCR4^+^ plasmablasts was significantly increased in Lgals3^−/−^ mice, corresponding to 4.3% of B220^+^CD138^+^ bone marrow cells in Lgals3^+/+^ mice and 18.1% of these bone marrow cells in Lgals3^−/−^ mice (Fig. 7D–F). Corroborating, the percentage of CD19^+^ B cells was markedly elevated in the blood of Lgals3^−/−^ mice and numerically three-fold higher in these mice, when compared with Lgals3^+/+^ mice (Fig. 7G–I). Moreover, the percentage of circulating B220^+^CD138^+^CXCR4^+^ plasmablasts was also increased in the absence of gal-3, corresponding to 1.5% of B220^+^CD138^+^ blood cells in Lgals3^+/+^ mice and 27.3% of these blood cells in Lgals3^−/−^ mice (Fig. 7J–L).

## Discussion

Gal-3 has been described as significant regulator of B cell differentiation^3,4,7,8^. However, the molecular and cellular mechanisms involved are poorly understood. Here, we demonstrated that gal-3 affects distinct time-points and niches of B cell differentiation into plasma cells. Lgals3^−/−^ mice showed important B cell disturbances frequently correlated with IL-7/IL-7R signaling in the bone marrow, Notch/Delta and Notch/Jagged in the spleen and CXCR4^+^ cell circulation in the blood. These data suggested that gal-3 inhibits B cell differentiation using complex mechanisms and possibly by tissue-specific dependent pathways.

In fact, gal-3 has multifunctional effects in the hematopoietic cells. The expression of gal-3 by myeloid cells is directly correlated with monocyte-macrophage and neutrophil functions^22,23^. In contrast, gal-3 inhibits conventional and peritoneal B cell differentiation^3,5^, suggesting that gal-3 is crucial to maintain an equilibrate hematopoiesis. In accordance, it is know that bone marrow hematopoiesis is predominantly myeloid in detriment to lymphoid development^24,25^. In this work, we have evidence pointing to cell-cell interactions between bone marrow stromal cells and B cell progenitors by IL-7 dependent manner as the major step regulated by gal-3. The high percentage of B220^low^CD19^low^c-Kit^+^IL-7Ra^+^ progenitor B cells was strongly correlated with abnormal gene expression by bone marrow stromal cells and the exacerbated mRNA levels of IL-7 justify the higher number of B cell progenitor in the bone marrow of these Lgals3^−/−^ mice.

IL-7 is essential to Pro-B cell subpopulation to proliferate during lymphoid development^26^. Consistently, IL-7^−/−^ and IL-7R^−/−^ mice have severe damages on B cell differentiation^27^ markedly characterized by extreme reduction in the number of Pro-B cells, Pre-B and differentiated B cells^28^. In this work, we reinforced these data when observed that B cell progenitors were significantly increased in the bone marrow of Lgals3^−/−^ mice, where stromal cells presented approximately 40 times greater mRNA IL-7 than bone marrow stromal cells of Lgals3^+/+^ mice. Moreover, methycellulose system revealed that B cell progenitors responded promptly to conditioned medium appropriate to expansion of IL-7R^+^ progenitors cells. In parallel, IL-7 transgenic mice produce approximately 50 times greater IL-7 than control mice. They have higher numbers of bone marrow progenitor B cells and persistent B cell differentiation in the spleen than WT mice^29^. For this reason, we suggested that Lgals3^−/−^ mice are phenocopy of IL-7 transgenic mice.

We also observed significant disorders in the spleen of Lgals3^−/−^ mice associated with abnormal B lymphocyte differentiation into plasma cells. The spleen is formed by well-defined histological zones: the white pulp and the red pulp, both separated by the marginal zone^30^. The marginal zone is considered an intriguing area composed of distinct cell types, including metallophilic macrophages, marginal zone macrophages, marginal zone B cells (MZ B cells), T lymphocytes, small B cells, and dendritic cells^31^. The presence of gal-3^+^ cells in strategic B cell niches indicates a close relationship between this lectin and splenic plasmacytogenesis. Although inconclusive, we found that gal-3 expressing cells within lymphoid follicles were predominantly elongated whereas in extra-follicular sites gal-3^+^ cells showed dendritic-like structures. In lymph nodes, a similar description was demonstrated during B cell lymphoma and chronic schistosomiasis^7,32^. A more specific morphological study needs to be done to correlate with cell functions and tissue organization.

In the absence of gal-3, the splenic marginal zone was severely disturbed and possibly correlated with the abnormal plasma cell generation in these mice. Marginal zone is a favorable microenvironment to contact phagocytic cells and systemic antigens due to low velocity of the bloodstream derived from marginal sinuses^31^. Here we demonstrated that gal-3^+^ macrophage-like cells delimit lymphoid follicles and red pulp, a histological region classically named as marginal zone. Hematoxilin & Eosin staining and immunohistochemistry to B220 clearly revealed that marginal zone was significantly affected by the absence of gal-3 and the limit between follicular B cell niches in the white pulp and plasma cell niches in the red pulp was uncertain. These data suggested that gal-3 favors the marginal zone organization in the spleen of adult mice and contributes with organized plasma cell generation *in vivo*.

Disorganization on cell niches in lymphoid organs is frequently associated with inappropriate tissue-response and several diseases, including lymphoid neoplasias, characterized by imbalances in the cell death index^33,34^. Lgals3^−/−^ mice showed an uncommon percentage of IgM^+^IgD^+^ in the bone marrow, blood and spleen, frequently higher than WT mice. This phenotype is attributed to splenic differentiation obtained by IgM^+^IgD^−^ immature B cells that move from the bone marrow to spleen, where terminally differentiate into B220^high^IgM^+^IgD^+^ mature B cells^35^. However, the existences of IgM^+^IgD^+^ B cells in the bone marrow of splenectomized mice indicate that these B cells can be differentiate in the bone marrow independently of the spleen^36^. To reinforce the importance of the spleen in our work, we investigated B220^+^CD138^+^CXCR4^+^ cells with phenotype similar to plasmablasts obtained in the spleen^37,38^ and we observed that B220^+^CD138^+^CXCR4^+^ cells were significantly increased in the blood and bone marrow of Lgals3^−/−^ mice. The hallmarked presence of these cells in the periferal blood and bone marrow of Lgals3^−/−^ mice strongly suggested a plasmablast expansion in the spleen reflecting preferentially in the bone marrow since it is a critical niche to plasma cell functions. These data suggested that disturbed splenic B cell differentiation in the absence of gal-3 was strictly associated with marginal zone disorganization, consequently affecting the quantitative equilibrium of circulating B cells.

We analyzed classic Notch ligand/receptors signaling pathways. In the bone marrow, stromal cells of Lgals3^−/−^ mice expressed higher levels of JAG1, a Notch ligand frequently associated with T lymphocyte precursors^39^. Our data indicated that JAG1 intensity of fluorescence was increased in the bone marrow stromal cells of Lgals3^−/−^, but not DLL1 and DLL4. Thus, we suggested that higher expression of JAG1 could be associated with expansion of IL-7R^+^ B cell progenitors in the absence of galectin-3. However, it is not clear whether galectin-3 controls directly the expression of JAG1 as well as its absence interferes with the modulation of JAG1 gene expression.

In the spleen, the expression of DLL1, DLL4 and JAG1 by stromal cells was significantly increased in the absence of gal-3. DLL1 enhances spontaneous Ig secretion by marginal zone B cells. Moreover, splenic expression of DLL1 and JAG1 were distinct in areas, indicating that Ig secretion is ligand-specific but critical to terminal B cell differentiation into plasma cells^15^. Moreover, these authors also demonstrated that expression of JAG1 was restricted to the marginal zone and few DLL1/ JAG1 double-expressing cells were detected. Thus, as we found that DLL1 expression was softly increased while JAG1 expression was intensively increased by splenic stromal cells of gal-3^−/−^ mice, we indicate Notch/JAG1 as the major molecular mechanism affected by the lack of gal-3 and possibly involved with abnormal plasma cell generation in the spleen of these knockout mice.

## Methods

### Animals

Eight-week-old Lgals3^+/+^ and Lgals3^−/−^ C57/Bl6 mice^40^ age and sex matched were obtained from the colony bred at the Federal University of Rio de Janeiro, Brazil. The experimental protocols involving mice were in accordance with guidelines provided by Brazilian College of Animal Experimentation (CONCEA - Conselho Nacional de Controle de Experimentação Animal) and approved by the Animal Ethics Committee (CEUA, Comissão de Ética no Uso de Animais) of Federal University of Rio de Janeiro, Brazil (number DAHEICB009).

### Bone marrow, blood and splenic cell suspensions

Bone marrow cells were obtained by flushing procedures. Briefly, femurs epiphysis was cut and bone cavity was washed with cold phosphate-buffered saline (PBS) and supplemented with 3% of fetal bovine serum (FBS). Blood cells were collected by cardiac puncture with a syringe containing no more than 10 IU of sodium heparin per mL of blood (Sigma, USA). The spleen cells were obtained by mechanic dissociation in phosphate buffer saline (PBS) and red blood cells were lysed in ammonium-chloride-potassium (ACK) solution, quantified and adjusted according to experimental condition.

### Bone marrow cell culture

Bone marrow cells of Lgals3^+/+^ and Lgals3^−/−^ mice were adjusted to 5.0 × 10^5^ cells in RPMI-1640 (Sigma-Aldrich, USA) supplemented with 10% Fetal Bovine Serum, 2 mM glutamine, 10^−5^ β-mercaptoethanol, and 100 mg/mL penicillin and streptomycin and maintained in 25 cm^2^ tissue culture flasks at 37 °C in 5% CO_2_ atmosphere. After 1 week in culture, adherent bone marrow cells were submitted to RNA extraction protocol as previously described^41^. To methylcellulose medium enriched with IL-7 (Methocult M3630, Stem Cell Technologies, Canada), 5 × 10^4^ total bone marrow cells were homogenously distributed in the flasks and maintained at 37 °C in 5% CO_2_ atmosphere for 1 week. The colonies (more than 50 cells) and clusters (less than 50 cells) of pre-B cells were quantified through inverted microscope Olympus CKX41SF (Olympus, Japan). The capture of images was performed using camera QColor-3 attached to the Q-Capture software (Olympus).

### Flow cytometry: phenotype and DNA content

Cell suspensions were adjusted to 1 × 10^6^ cells/mL and Fc receptors blocked (Fc blocker antibody, clone 2.4G2) for 10 min. The following monoclonal antibodies were used for membrane staining: anti-B220, CXCR4, Mac-1 (streptoavidin FITC); anti-CD19, IL7Ra, CD138 phycoerithrin (PE); anti-CD45, B220 phycoerithrin Cychrome (PECy5.5); anti-IL-7Ra, IgM (Biotin-Streptoavidin PerCP) and anti-c-Kit, IgD alophicocianin (APC). IgG isotype control FITC. All monoclonal antibodies were purchased from BD Bioscience, USA. Bone marrow cells and splenic cells from Lgals3^+/+^ and Lgals3^−/−^ mice were labeled with anti-CD45 (pan-leukocyte marker) for identification of stromal cells (CD45^−^ cells). The anti-DLL1, DLL4 and JAG1 monoclonal antibodies (Santa Cruz, USA) were submitted to intracellular staining as described previously^42^. DNA-content was measured by propidium iodide labeling using Vindelov solution^43^. Samples were acquired in FACScalibur, flow cytometer (BD Bioscience), using software Cell Quest and analyzed in both Cell Quest and WinMDI 2.9. All experimental procedures with flow cytometry were performed using 5 mice per group in 3 independent experiments.

### Cell sorting

Total spleen cells from Lgals3^+/+^ and Lgals3^−/−^ mice were treated with ACK solution for red blood cells lysis and subsequently stained with monoclonal anti-B220 APC. Total B220^ +^ cells were sorted to perform western blotting analysis using FacsAria2 cytometer (BD Bioscience) with 98% of purity.

### Histological analysis

For histological analysis, the spleen of Lgals3^+/+^ and Lgals3^−/−^ mice were removed, processed and cut into 0.5 mm-thick slices, washed in cold PBS and fixed in 10% buffered formalin for 12 hours. After this period, specimens were dehydrated in alcohol and embedded in paraffin. Sections of 5 µm were obtained and stained with hematoxylin & eosin. For the immunohistochemistry procedures, paraffin-embedded sections were de-waxed and hydrated. After inhibition of endogenous peroxidase, sections were incubated for 1 h with 0.01 M PBS containing 5% BSA, 4% skim milk, 0.1% Triton X-100 (Sigma Aldrich, USA), 0.05% Tween-20, and 10% normal goat serum and incubation with the following purified antibodies: anti-gal-3 (clone M3/38; American Type Culture Collection, USA, at 1∶10 in PBS, 3% BSA and 1% normal goat serum), anti-B220 and anti-CD138 (Santa Cruz Biotechnology, USA) overnight at 4 °C in a humid chamber. Antibodies were detected with a biotinylated anti-rat IgG (BA-4001, Vector Laboratories, USA) and developed with avidin-peroxidase (1∶50 in PBS) (Sigma Aldrich, USA), using diaminobenzidyne as the chromogen. Sections were counterstained with Harris’ hematoxylin. Bright-field pictures were acquired using an Evolution MP 5.0 RTV Color camera (Media Cybernetics, Canada). As negative controls, sections of Lgals3^+/+^ and Lgals3^−/−^ mice tissue were incubated with non-immune rat serum instead of anti-gal-3 antibody.

### Real-time quantitative PCR analysis

Bone marrow and spleen stromal cells were trypsinized and adjusted to 1 × 10^6^ cells/mL. After wash in PBS, pellet was submitted to RNA extraction using the TRIzol reagent (Invitrogen Life Technologies, USA), following the manufacturer’s instructions. cDNA synthesis was performed in a final volume of 20 μL, using ImProm-II reverse transcriptase (Promega Corporation, USA). The reaction mixture contained 4 μg total RNA, 20 pmol oligo dT primer (Invitrogen Life Technologies), 40 U RNasin, 500 μM of dNTP mix, and 1 U reverse transcriptase in the 1× reverse transcriptase buffer. The cDNA was treated with 10 μg RNase (Gibco, USA) and then immediately used or stored at −20 °C. PCR amplification and analysis were done using an ABI Prism 7500 sequence detector (Applied Biosystems, USA). All the reactions were done with SYBR Green Master Mix (Applied Biosystems) using a 25 μL volume in each reaction, which contained 2 μL template cDNA, 5 pmol each primer, and 12.5 μL SYBR Green. The relative expression of each gene was obtained using the comparative CT method, and they were normalized using β-actin as an endogenous control. Therefore, data in graphs of relative expression represent the ratio between the mRNA levels of the target gene and the expression of β-actin. Primers are listed on Table 3.

**Table 3:** Primers used for reverse transcriptase polymerase chain reaction (RT-PCR).

Gene	Acess Number	Sequence
β-actin	NM 007393	F: 5′-CCTAAGGCCAACCGTGAAAA-3′
		R: 5′-GAGGCATACAGGGACAGCACA-3′
Blimp-1	NM 007548	F: 5′-AAGTCTAGCTCCGGCTCCGT-3′
		R: 5′-TCGGCCTCTGTCCACAAAGT-3′
IL-4	NM 021283	F: 5′-GTCTCTCGTCACTGACGGCA-3′
		R: 5′-CGTGGATATGGCTCCTGGTAC- 3′
IL-5	NM 010558	F:5-AGACGGAGGACGAGGCAGTT-3′
		R: 5′-CACCATGGAGCAGCTCAGC 3′
IL-7	NM 008371	F: 5′-CTTGCTTTTTCCAGCCACGT-3′
		R: 5′-AGGCATGGCTACCACACATG-3′
GM-CSF	NM 009969	F: 5′-CCTGGGCATTGTGGTCTACAG-3′
		R: 5′-GGTTCAGGGCTTCTTTGATGG-3′
M-CSF	NM 007778	F: 5′-GTGAGCTTCCCCTTCGCATA-3′
		R: 5′-CCCATGGTTTGGTTGCTCTG-3′
DLL1	NM 007865	F: 5′-TTCTTTCGCGTATGCCTCAA-3′
		R: 5′-AAGGAGTCGACACCCAGCAC-3′
DLL4	NM_019454.3	F: 5′-AGGTGCCACTTCGGTTACAC-3′
		R:5′-GGGAGAGCAAATGGCTGATA-3′
JAG1	NM_013822.5	F: 5′-TGGCCGAGGTCCTACACTT-3′
		R: 5′-GCCTTTTCAATTATGCTATCAG-3′
HEY1	NM_010423.2	F:5′-CATGAAGAGAGCTCACCCAGA-3′
		R:5′-CGCCGAACTCAAGTTTCC-3′

### Immunoblotting

Cells were lysed in RIPA buffer and 50 µg of proteins were separated by Novex NuPAGE SDS-PAGE gel system (Invitrogen). The membrane was incubated with anti-cleaved Val1744 (NICD1) (Cell signaling). Anti-β-actin-peroxidase was used as a loading control. Horseradish peroxidase (HRP)-conjugated antibodies were detected using the enhanced chemiluminescence (ECL) reagent (GE Healthcare). Splenic cells were sorted according B220 surface expression. Four mice per group (Lgals3^+/+^ and Lgals3^−/−^) were used to FACS isolation and approximately 4 × 10^6^ cells positive to B220 were recovered per mouse (individually).

### Statistical analysis

The statistical tests were accomplished using Tukey’s multiple comparison test (t-test), and significance threshold was fixed for α = 0.05. Therefore, P values ≤ 0.05 were considered statistical differences. Each experiment was performed using 3–5 mice per group in three independent essays.

## Electronic supplementary material

Supplementary Figures
