# Supplementary material for: Lack of galectin-3 modifies differentially Notch ligands in bone marrow and spleen stromal cells interfering with B cell differentiation
Source: Sci Rep. 2018 Feb 22;8:3495. doi: 10.1038/s41598-018-21409-7 (PMC5823902; doi:10.1038/s41598-018-21409-7)
Supplement: Supplementary file 1 — Supplementary Figures [file 41598_2018_21409_MOESM1_ESM.pdf]

Lack of galectin-3 modifies differentially Notch ligands in bone marrow and spleen stromal cells interfering with B cell differentiation

Felipe Leite de Oliveira, Sofia Nascimento dos Santos, Lauremília Ricon, Thayse Pinheiro, Jonathas Xavier Pereira, Camila Brand, Marise Lopes Fermino, Roger Chammas, Emerson Bernardes, and Márcia Cury El-Cheikh.

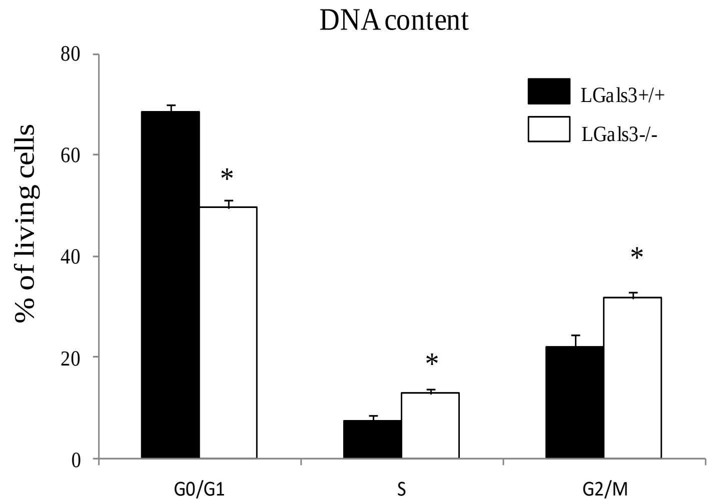

Supplementary Figure 1: DNA content in the bone marrow hematopoietic cells. Cell cycle phases were monitored by flow cytometry. “G0/G1” indicates diploid cells. “S” represents phase of DNA synthesis corresponding to cells with growing DNA content. “G2/M” indicates cells with DNA totally duplicated and prepared to duplication (mitotic phase). Black bars indicate wild type (WT) mice and white bars indicate galectin-3 knockout mice (Gal-3$^{-/-}$). *n*=5 mice per group. (*) Indicates $p<0.05$.

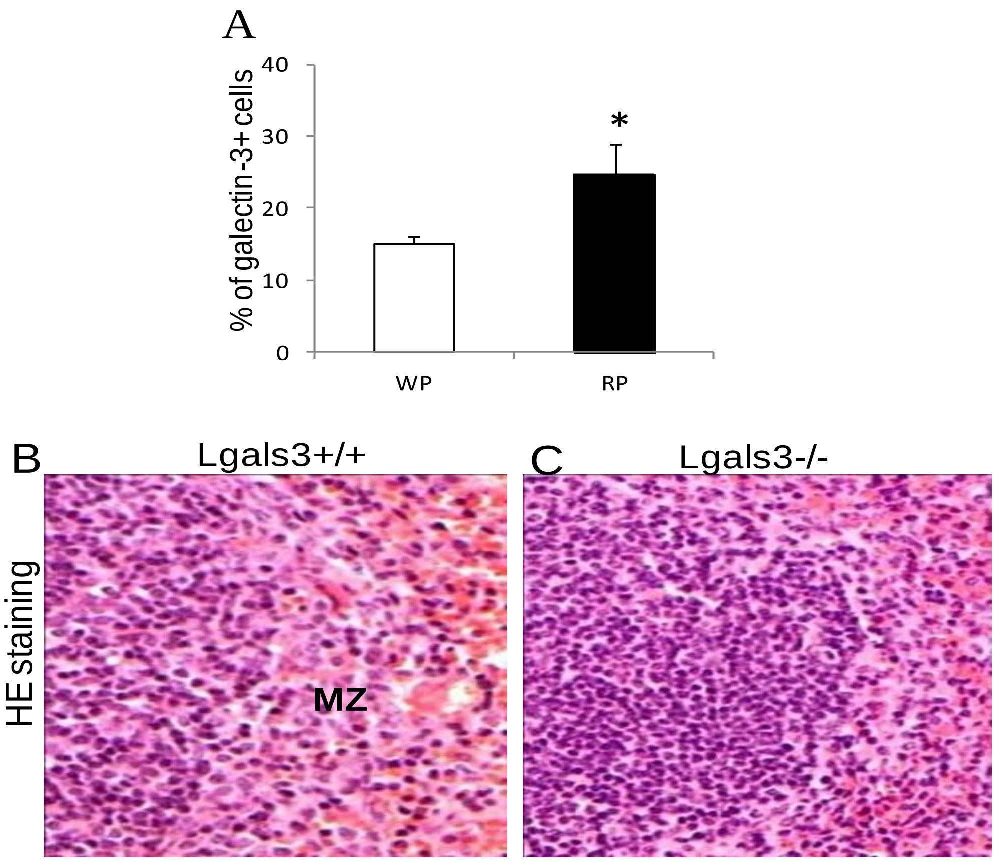

Supplementary Figure 2: Morphological analysis of marginal zone in the spleen of Lgals3$^{-/-}$ mice. (A) Quantitative representation of splenic cells expressing gal-3. Heamatoxilin and eosin staining reveal classic organization of marginal zone on Lgals3$^{+/+}$ mice (B), but substantially disorganized in Lgals3$^{-/-}$ mice (C). Magnification: 200x; *n*=5 mice per group. (*) Indicates $p<0.05$.
